# Intervention versus standard medical treatment in patients with symptomatic occlusion of the internal carotid artery: a randomised oxygen-15 PET study

**DOI:** 10.1186/2191-219X-3-79

**Published:** 2013-12-05

**Authors:** Suzanne Persoon, Bart NM van Berckel, Jochem P Bremmer, Ronald Boellaard, Ale Algra, Gert Jan de Borst, Adriaan A Lammertsma, L Jaap Kappelle, Catharina JM Klijn

**Affiliations:** 1 Department of Neurology and Neurosurgery, Rudolf Magnus Institute of Neuroscience, University Medical Center Utrecht, G03.228, Heidelberglaan 100, Utrecht 3584 CX, The Netherlands; 2 Department of Nuclear Medicine & PET research, VU University Medical Center, De Boelelaan 1117, Amsterdam 1081 HV, The Netherlands; 3 Department of Epidemiology, Julius Center for Health Sciences and Primary Care, University Medical Center Utrecht, Heidelberglaan 100, Utrecht 3584 CX, The Netherlands; 4 Department of Vascular Surgery, University Medical Center Utrecht, Utrecht 3584 CX, The Netherlands

**Keywords:** Carotid artery diseases, Haemodynamics, Other cerebrovascular disease/stroke, PET

## Abstract

**Background:**

The aim of this randomised pilot study was to investigate the haemodynamic effects measured by oxygen-15 positron emission tomography (PET) of interventional treatment consisting of either endarterectomy or endovascular treatment of stenosed cerebropetal arteries, or tapering of antihypertensive medication in comparison with standard medical treatment alone in patients with symptomatic internal carotid artery (ICA) occlusion.

**Methods:**

Twenty-three patients with symptomatic ICA occlusion underwent PET scanning at baseline and after 3 months. Twelve patients were randomised to intervention (either endarterectomy or endovascular treatment of stenosed cerebropetal arteries, or tapering of antihypertensive medication) and 11 to standard medical treatment alone. Primary outcome was a change in cerebral blood flow (CBF), cerebral blood volume (CBV) and/or oxygen extraction fraction (OEF) after 3 months measured by PET.

**Results:**

There were no differences in changes in CBF, CBV or OEF between the two groups. Only patients with compromised perfusion at presentation showed a borderline significant increase in CBF of 2.8 mL/min/100 mL (95% confidence interval 0.0 to 5.7) after intervention (*n* = 7).

**Conclusion:**

This pilot study shows that in patients with symptomatic ICA occlusion, oxygen-15 PET did not detect differences in improvement of CBF, CBV or OEF between interventional and standard treatment.

## Background

In patients with transient ischaemic attack (TIA) or minor disabling ischaemic stroke associated with an occlusion of the internal carotid artery (ICA), the risk of recurrent stroke has been reported 5% to 6% per year [1]. This risk may be as high as 12% per year in case of a demonstrated compromised flow to the brain [2-4]. In these ‘high-risk’ patients, revascularization surgery may be considered, but evidence that interventional treatment reduce the risk of stroke in comparison with the standard treatment consisting of antithrombotic medication and control of vascular risk factors is lacking.

A recent study has shown that increased oxygen extraction fraction (OEF) measured by positron emission tomography (PET) is still an independent predictor of subsequent stroke under current medical treatment [5]. However, the Carotid Occlusion Surgery Study (COSS) included patients with symptomatic ICA occlusion and poor cerebral haemodynamics as demonstrated by increased OEF measured by PET and found no benefit of extracranial/intracranial (EC/IC) bypass surgery [4,6]. This trial was stopped prematurely as the 2-year risk of ipsilateral stroke was between 20% and 25% in both surgical and non-surgical groups (*p* = .78). In this study, the proportion of patients who had a perioperative stroke (within 30 days after surgery) was 15% [4].

Other therapeutic strategies in patients with symptomatic ICA occlusion aiming to improve cerebral perfusion and reducing the risk of recurrent stroke are endarterectomy or endovascular treatment of a significant stenosis in one of the cerebropetal arteries that are part of collateral pathways [2,7-10], or tapering of antihypertensive medication [11,12].

These procedures have lower perioperative risks than an EC/IC bypass operation, but so far, their clinical effect has not been compared to standard treatment. Whether the application of these therapeutic strategies improves cerebral perfusion as compared with standard medical treatment is unclear.

The aim of this randomised pilot study was to investigate the haemodynamic effects as measured by oxygen-15 PET of endarterectomy or endovascular treatment of stenosed cerebropetal arteries, or tapering of antihypertensive medication in comparison with the standard medical treatment alone in patients with a symptomatic ICA occlusion.

## Methods

### Patients

Between December 2004 and September 2009, patients who had been referred to the University Medical Center Utrecht with a symptomatic ICA occlusion were considered for participation. Inclusion criteria were (1) transient or at most, moderately disabling (modified Rankin scale ≤ 3 [13]) neurological deficits associated with ischaemia in the hemisphere ipsilateral to the ICA occlusion, (2) ICA occlusion proven by angiograph and (3) symptoms present within the previous 3 months. Exclusion criteria were (1) ICA occlusion caused by arterial dissection or radiation vasculopathy, (2) contra-indications for magnetic resonance imaging (MRI) (claustrophobia or metal objects in the body) or PET (pregnancy, blood donation in the previous 3 months or haemoglobin concentration <8.0 mmol/L) and (3) absence of any of the conditions that could be treated according to the therapeutic strategy (see below). All patients were interviewed regarding symptoms and risk factors as listed in Table 1, underwent neurological examination, measurement of blood pressure and blood tests for glucose and lipids. Contrast angiography was performed to confirm occlusion of the ICA by absence of filling of the extracranial ICA, to assess collateral blood supply to the symptomatic hemisphere, and the presence of stenosis in the cerebropetal arteries [14].

**Table 1:** **Clinical characteristics and angiographic findings of patients with symptomatic occlusion of the internal carotid artery (*n*** **= 23)**

	**Intervention *n*** **= 12**	**Standard *n*** **= 11**
Age (years, mean ± SD)	68 ± 10	60 ± 12
Male	12	7
Clinical features at presentation		
Cerebral TIA	9	8
Ischaemic stroke	3	3
Systolic blood pressure (mmHg, mean ± SD )	159 ± 25	167 ± 29
Diastolic blood pressure (mmHg, mean ± SD)	84 ± 15	91 ± 13
Vascular risk factors		
Hypertension^a^	10	10
Hyperlipidaemia^b^	10	10
Diabetes mellitus	2	3
Cigarette smoking (current or in last 5 years)	7	7
History of stroke >3 months ago	3	4
History of ischaemic heart disease	2	6
Angiogram		
Contralateral ICA stenosis 50% to 69%	5	2
Stenosis 70% to 99%	1	2
Occlusion	0	1
Ipsilateral ECA stenosis ≥ 50%	4	1
Vertebral artery stenosis ≥ 50%	5	5
Collateral flow via anterior communicating artery	12	10
Collateral flow via ophthalmic artery	5	5
Collateral flow via posterior communicating artery	9^c^	11
Leptomeningeal collaterals	7^d^	9

^a^Blood pressure > 160/95 mmHg or the current use of antihypertensive medication; ^b^patients with either a history of hyperlipidaemia, patients on statins or patients with levels of total cholesterol, triglycerids or high density lipoprotein cholesterol outside normal ranges; ^c^the presence of collateral flow via the posterior communicating artery could not be judged in one patient; ^d^the presence of leptomeningeal collaterals could not be judged in two patients.

Patients were randomised between any intervention according to therapeutic strategy or standard medical treatment. In the intervention group, the therapeutic strategy consisted of, in order of preference, one of the following treatment options: (1) endarterectomy (CEA) of the contralateral ICA in case of a severe contralateral ICA stenosis in the presence of a functional anterior circle of Willis, (2) CEA of the external carotid artery (ECA) in case of a severe ipsilateral ECA stenosis in the presence of collateral blood supply via the ECA and ophthalmic artery, (3) endovascular treatment of the vertebral artery (VA) or subclavian artery in case of a severely stenosed VA or subclavian artery in the presence of collaterals via the vertebrobasilar system and (4) tapering of antihypertensive medication for 3 months. In addition to the intervention, patients received standard medical treatment consisting of antithrombotic medication (aspirin and dipyridamole), a statin, treatment of hypertension to a targeted blood pressure of 140/90 mmHg (except for those randomised to tapering of antihypertensive drugs) and control of other vascular risk factors such as smoking and obesity. To ensure equal distribution of patients with a low CBF in both groups [15], randomisation was carried out by minimisation for CBF in three categories (CBF ≤ 33 mL/100 g/min, CBF between 34 and 53 mL/100 g/min or CBF ≥ 54 mL/100 g/min), measured in the MCA territory of the symptomatic hemisphere by arterial spin labelling (ASL)-MRI [16]. The ASL-MRI scan was made within 1 to 15 days of the baseline PET study, and included a three-dimensional (3D) T1 image for PET image analysis. Patients were examined by oxygen-15 PET at the time of inclusion and 3 months thereafter. Primary outcome was a change in CBF, CBV and/or OEF as measured by PET after 3 months. During those 3 months, we documented whether patients had recurrent TIA or stroke, defined as an acute onset of transient (<24 h) or permanent new focal neurological deficits of cerebral origin without haemorrhage on CT or MRI.

The institutional medical review board approved the study protocol, and all patients provided written informed consent.

### PET data acquisition

Oxygen-15 PET scans were acquired using an ECAT EXACT HR + scanner (CTI/Siemens, Knoxville, TN, USA) [17]. Each PET study consisted of three parts: (1) a dynamic emission scan (25 frames over 600 s) after intravenous administration of a bolus of 1,100 MBq [^15^O]H_2_O to measure CBF; (2) a dynamic emission scan (20 frames over 600 s) after a 30-s net inhalation of approximately 300 MBq [^15^O]O_2_ gas through a nasal cannula to derive oxygen consumption and calculate OEF; (3) an emission scan (3 frames over 360 s) following a net inhalation of approximately 200 MBq [^15^O]CO gas to measure CBV. Further details of the scanning procedure can be found elsewhere [18].

### Image analysis

Individual anatomical 3D T1 MR images were co-registered with summed [^15^O]H_2_O images. A standard template of flow territories of middle cerebral artery (MCA), anterior cerebral artery (ACA) and posterior cerebral artery (PCA) [19] was warped onto the co-registered MR image using Automated Image Registration (AIR) software [20], applying non-rigid 12 parameter perspective warping. Statistical Parametric Mapping (SPM02, London, UK, application in Matlab 7.0.4; MathWorks, Inc., Natick, MD, USA) was used for segmentation of grey and white matter. Areas of infarction were excluded manually. Parametric CBF, CBV and OEF images were generated using in-house developed software (written in IDL, 6.2, ITT, Boulder CO, USA) [21].

### Data analysis

Normal CBF, CBV and OEF values were derived from 14 scans in seven healthy subjects (mean age 66 ± 7 years; five men). They were all in a good physical and mental condition, which had been evaluated with medical history, a physical examination and screening laboratory tests. Subjects with a history of psychiatric or neurological illness, a Mini Mental State Examination score ≤ 27 or any significant abnormality of a laboratory test were excluded. They underwent a PET scan on two separate occasions with a median time interval of 7 days, as published previously [18]. CBF, CBV and OEF values in patients were considered to be abnormal if they were beyond mean values of normal controls ±1.96 times the standard deviation (SD). Patients were divided into haemodynamic stages based on their values in grey matter of the affected MCA territory: patients with normal CBF (≥31.1 mL/min/100 mL), normal CBV (≤3.9 mL/100 mL) and normal OEF (≤55.7%) were classified in haemodynamic stage 0, patients with either decreased CBF (<31.1 mL/min/100 mL) or increased CBV (>3.9 mL/100 mL), but normal OEF in haemodynamic stage 1, and patients with increased OEF (>55.7%) in haemodynamic stage 2.

We compared mean CBF in the MCA territory of patients with data in healthy subjects [18] and calculated a mean difference with 95% confidence interval (CI). In the patients, differences between mean baseline grey matter CBF, CBV and OEF values in ACA and MCA territories of patients at baseline and after 3 months were assessed by paired *t* tests with 95% confidence interval (CI). Mean absolute change, adjusted for age and sex, in CBF, CBV and OEF was compared between patients assigned to intervention and those who received standard treatment using linear regression analysis, and results were expressed as adjusted mean differences in change with 95% CI. Finally, pre-specified subgroup analyses were performed in patients with haemodynamic stage 1 or 2 at presentation and between patients who were randomised to treatment with surgery or stenting of a stenosis in a collateral artery (excluding those who had tapering of antihypertensive medication) and those who received standard medical treatment.

## Results

**Figure 1:**
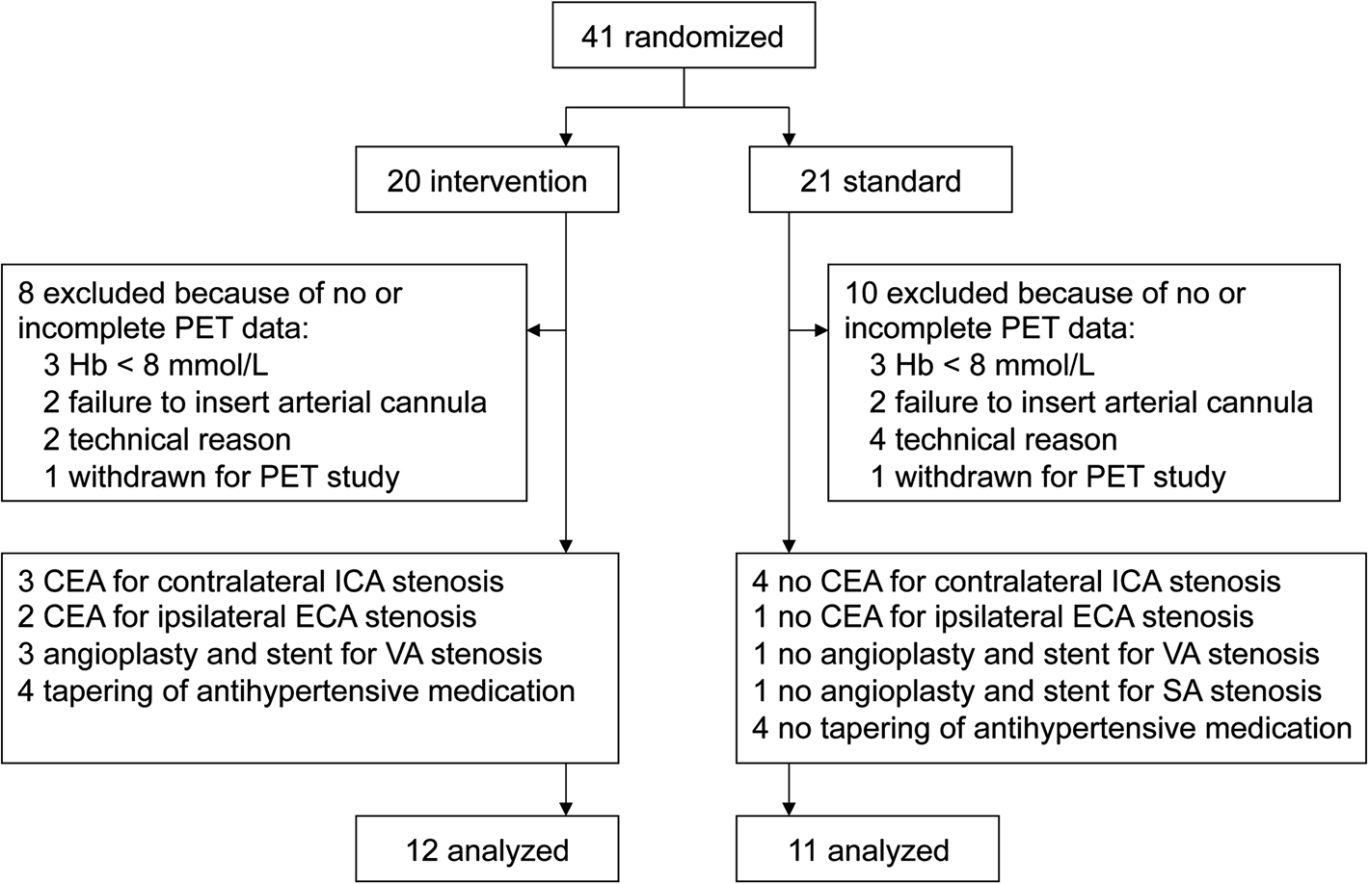
**Trial profile.** CEA, carotid endarterectomy; ICA, internal carotid artery; ECA, external carotid artery; VA, vertebral artery; SA, subclavian artery.

**Figure 2:**
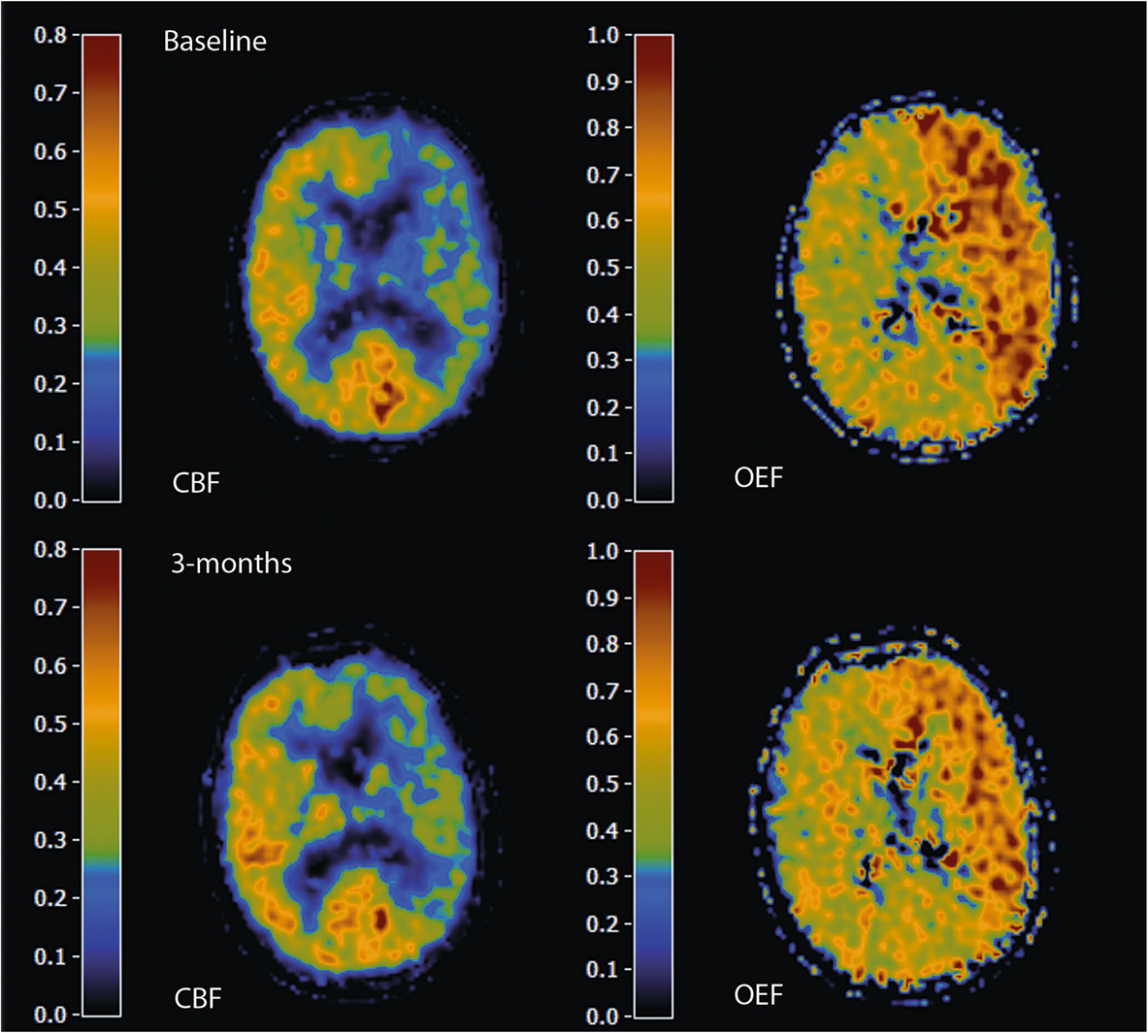
**Parametric images of a patient with stage 2 haemodynamic failure.** This 69-year-old man presented with a minor ischaemic stroke (without infarction on his MRI) and recurrent TIAs from the left hemisphere associated with a left ICA occlusion. The baseline PET study showed decreased cerebral blood flow (CBF) and increased oxygen extraction fraction (OEF) in the territories of both anterior and middle cerebral artery of the left hemisphere. This patient was randomised for tapering of antihypertensive medication. The PET study after 3 months showed a slight improvement in CBF and OEF. However, he still had recurrent TIAs in the presence of stage 2 haemodynamic failure.

Complete PET data sets were obtained for 23 patients (mean age 64 ± 11 years; 19 men). Of 41 eligible patients, 14 failed to complete the initial PET study and were excluded and four others did not complete the PET study after 3 months (Figure 1). Twelve patients were assigned to intervention (eight stenting or surgery and four tapering of antihypertensive medication) and 11 to standard treatment. Clinical characteristics and presence of stenosis in cerebropetal arteries and collateral pathways are shown in Table 1. Twenty-two patients had had recurrent ischaemic symptoms after the ICA occlusion had been demonstrated, but before inclusion in the study. The time between last symptoms and first PET scan was on average 38 ± 23 days in both treatment groups. At baseline, ten patients were characterized as haemodynamic stage 0, 11 as stage 1 and two as stage 2. Figure 2 shows parametric images of a typical patient with stage 2 haemodynamic failure. Patients with a symptomatic ICA occlusion had a mean CBF in the ipsilateral MCA territory of 32.2 ± 5.6, which was significantly lower (mean difference −5.2 mL/min/100 mL, 95% CI −9.8 to −0.6) than that in healthy subjects (mean CBF MCA territory of 37.4 ± 3.2) [18].

The mean time between PET scans at baseline and follow-up was 98 ± 10 (range 77 to 119) days. All patients were treated according to their randomisation. Randomisation occurred on average 5 ± 9 days after the baseline PET scan. Of the 12 patients assigned to intervention (Figure 1), eight patients underwent stenting or surgery after a mean time of 15 ± 10 (range 1 to 29) days following the baseline PET scan. In four patients, antihypertensive medication was tapered, which resulted in a mean increase in systolic blood pressure of 38 ± 9 mmHg. Intervention was complicated by a non-fatal myocardial infarction in one patient who underwent CEA of a contralateral ICA stenosis; none of the procedures were complicated by a stroke or hyperperfusion syndrome.

**Figure 3:**
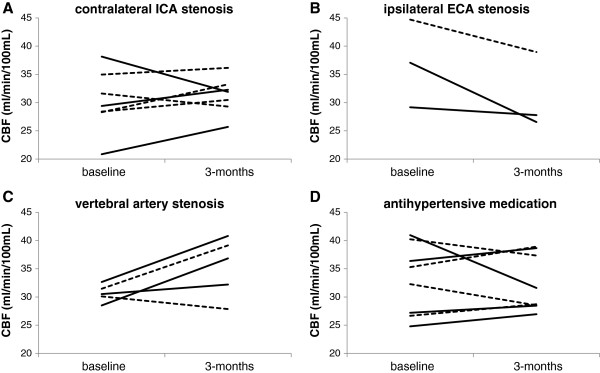
**CBF changes over time in the MCA territory for individual patients.** Patients randomised to **(A)** CEA of a contralateral ICA stenosis, **(B)** CEA of an ipsilateral ECA stenosis, **(C)** angioplasty and stent placement of a VA stenosis and **(D)** tapering of antihypertensive medication. Patients randomised to intervention are indicated by continuous lines and patients randomised to standard treatment by dashed lines.

Mean values of CBF, CBV and OEF were similar at baseline and after 3 months, both in patients treated with intervention and in those assigned to standard treatment (Table 2), but CBF changes varied in individual patients (Figure 3). No differences in absolute CBF, CBV and OEF changes after 3 months were observed between patients assigned to intervention and those assigned to standard treatment (Table 2).

**Table 2:** **Comparison of haemodynamic state of the brain at baseline and after 3 months between patients assigned to intervention and those to standard treatment (*n*** **= 23)**

		**Intervention *n*** **= 12**	**Standard *n*** **= 11**	**Unadjusted difference in change (95% CI)**	Adjusted difference in change^* ^**(95% CI)**
		**Mean ± SD**	**Mean ± SD**		
CBF MCA (mL/min/100 mL)	Baseline	31.3 ± 5.9	33.1 ± 5.4		
	3 months	31.6 ± 4.9	33.5 ± 4.7		
	Change	0.34 ± 6.2	0.42 ± 4.1	−0.1 (−4.7 to 4.5)	−1.0 (−7.4 to 5.4)
CBF ACA (mL/min/100 mL)	Baseline	32.6 ± 5.8	35.2 ± 5.5		
	3 months	32.0 ± 4.2	35.0 ± 5.7		
	Change	−0.56 ± 6.2	−0.28 ± 4.9	−0.3 (−5.2 to 4.6)	−1.6 (−8.4 to 5.2)
CBV MCA (mL/100 mL)	Baseline	3.0 ± 0.6	3.3 ± 0.9		
	3 months	3.5 ± 1.4	3.2 ± 0.6		
	Change	0.60 ± 1.7	−0.09 ± 1.2	0.7 (−0.6 to 2.0)	0.09 (−1.7 to 1.9)
CBV ACA (mL/100 mL)	Baseline	3.0 ± 0.6	3.4 ± 1.0		
	3 months	3.5 ± 1.3	3.1 ± 0.7		
	Change	0.49 ± 1.6	−0.23 ± 1.3	0.7 (−0.6 to 2.0)	0.08 (−1.6 to 1.8)
OEF MCA (%)	Baseline	46.4 ± 9.7	47.5 ± 5.5		
	3 months	46.3 ± 7.9	48.4 ± 5.8		
	Change	−0.08 ± 6.1	0.85 ± 6.8	−0.9 (−6.5 to 4.7)	3.1 (−4.1 to 10.3)
OEF ACA (%)	Baseline	44.4 ± 11.2	46.1 ± 6.2		
	3 months	44.2 ± 7.3	47.2 ± 5.2		
	Change	−0.17 ± 7.4	1.1 ± 6.3	−1.3 (−7.3 to 4.7)	3.1 (−4.6 to 10.8)

Asterisk ‘*’ indicates adjusted for age and sex; measurements were performed ipsilateral to the side of the ICA occlusion; CBF, cerebral blood flow; CBV, cerebral blood volume; OEF, oxygen extraction fraction; MCA, middle cerebral artery; ACA, anterior cerebral artery.

Of the ten patients with normal CBF, CBV and OEF at baseline (haemodynamic stage 0), five underwent interventional treatment (CEA of the contralateral ICA in one, CEA of the ipsilateral ECA in one, stenting of the VA in one, and tapering of antihypertensive medication in two patients), which did not result in a further increase in CBF in the MCA territory (mean difference −3.1, 95% CI −13.1 to 6.9, Table 3). Of the 13 patients with haemodynamic compromise (haemodynamic stage 1 or 2), five underwent stenting or surgery (CEA of the contralateral ICA in two, CEA of the ipsilateral ECA in one, and stenting of the VA in two patients), in two, the antihypertensive medication was tapered and six received standard treatment. The seven patients assigned to intervention had a mean CBF in the MCA territory of 27.2 ± 3.4 mL/min/100 mL at baseline, which increased to 30.0 ± 3.9 mL/min/100 mL after 3 months (mean difference 2.8 mL/min/100 mL, 95% CI 0.0 to 5.7). The mean increase in CBF in the MCA territory in patients treated by intervention was, however, not significantly different from the increase in CBF in patients who received standard treatment (Table 3). Mean CBV in the MCA territory in patients with haemodynamic compromise treated with an intervention showed a small non-significant increase but did not differ from the change in CBV in patients who received standard treatment (Table 3). Because only two patients had an increased OEF at baseline, the effect of intervention on mean OEF after 3 months could not be formally compared. One of the two patients was assigned to standard treatment and after 3 months OEF was unchanged. In the other patient antihypertensive medication was tapered and OEF decreased from 72% to 66% after 3 months.

**Table 3:** **Mean CBF and CBV for patients with symptomatic ICA occlusion, classified in haemodynamic stage 0 (*n*** **= 10) and haemodynamic stage 1 or 2 (*n*** **= 13)**

		**Intervention**	**Standard**	**Unadjusted difference in change (95% CI)**	Adjusted difference in change^* ^**(95% CI)**
		**Mean ± SD**	**Mean ± SD**		
Haemodynamic stage 0		*N* = 5	*N* = 5		
CBF MCA (mL/min/100 mL)	Baseline	37.0 ± 3.0	35.0 ± 5.6		
	3 months	33.9 ± 5.8	34.4 ± 5.2		
	Change	−3.1 ± 8.1	−0.6 ± 5.3	−2.5 (−12.5 to 7.4)	−1.7 (−15.6 to 12.2)
CBV MCA (mL/100 mL)	Baseline	3.1 ± 0.6	2.8 ± 0.7		
	3 months	3.0 ± 0.7	3.5 ± 0.4		
	Change	−0.2 ± 1.1	0.6 ± 1.0	−0.8 (−2.3 to 0.7)	−1.1 (−3.2 to 1.1)
Haemodynamic stage 1 or 2		*N* = 7	*N* = 6		
CBF MCA (mL/min/100 mL)	Baseline	27.2 ± 3.4	31.5 ± 5.2		
	3 months	30.0 ± 3.9	32.8 ± 4.6		
	Change	2.8 ± 3.1	1.3 ± 3.2	1.6 (−2.2 to 5.4)	−1.1 (−7.8 to 5.7)
CBV MCA (mL/100 mL)	Baseline	2.9 ± 0.6	3.7 ± 0.8		
	3 months	4.0 ± 1.7	3.0 ± 0.6		
	Change	1.1 ± 1.9	−0.7 ± 1.1	1.8 (−0.2 to 3.8)	1.4 (−2.4 to 5.1)

Asterisk ‘*’ indicates adjusted for age and sex; CBF, cerebral blood flow; CBV, cerebral blood volume; MCA, middle cerebral artery.

The eight patients treated with surgery or stenting of a stenosis in a collateral artery had a mean CBF of 30.8 ± 5.4 mL/min/100 mL at baseline and 31.8 ± 5.2 mL/min/100 mL after 3 months (mean difference 0.97, 95% CI −4.6 to 6.6). In the 11 patients who received standard medical treatment alone, mean CBF was 33.1 ± 5.4 mL/min/100 mL at baseline and 33.5 ± 4.7 mL/min/100 mL after 3 months (mean difference 0.42, 95% CI −2.4 to 3.2). The change in CBF did not differ between these two groups of patients (adjusted mean difference of change −0.02 mL/min/100 mL, 95% CI −7.2 to 7.1).

### Clinical outcome

During the period between PET scans at baseline and after 3 months, 10 (43%) of 23 patients had one or more recurrent TIAs and none had a stroke. At baseline, five of the ten patients were in haemodynamic stage 0, three in haemodynamic stage 1, and two in haemodynamic stage 2. Two of the eight patients (25%) who underwent CEA or stenting, three of the four (75%) who had tapering of antihypertensive medication and 5 of the 11 (45%) who received standard medical treatment had one or more recurrent TIAs during the 3 months follow-up period between PET scans at baseline and after 3 months. Clinical follow-up continued with an overall median duration of 2.9 (range 0.5 to 4.4) years. Three of the 23 patients (13%) had a recurrent ischaemic stroke during follow-up.

The first patient experienced multiple TIAs and a minor ischaemic stroke (modified Rankin scale 2) in the hemisphere ipsilateral to the ICA occlusion 47 days after CEA of the contralateral ICA. The second patient had been treated with CEA of the ipsilateral ECA. In this patient, CBF was 37.1 mL/min/100 mL at baseline, which deteriorated to 26.6 mL/min/100 mL after 3 months. Because of ongoing TIAs, he also underwent CEA of the contralateral ICA 5 months after the 3-month PET scan. Nine months later, he had a fatal ischaemic stroke in the hemisphere ipsilateral to the ICA occlusion. The third patient was randomised for standard treatment (not tapering antihypertensive medication). In this patient, CBF was 32.3 mL/min/100 mL at baseline, deteriorating to 28.5 mL/min/100 mL after 3 months. He continued to have TIAs during the period between the two PET scans and thereafter and underwent an uncomplicated EC/IC bypass operation 9 months after randomization. Three months thereafter, he had a fatal ischaemic stroke in the hemisphere contralateral to the ICA occlusion.

## Discussion

This study shows that patients with recent symptoms of cerebral ischaemia associated with occlusion of the ICA on average have a lower CBF in the hemisphere ipsilateral to the occlusion than healthy subjects. Oxygen-15 PET showed that interventions aimed at improving cerebral perfusion caused a borderline significant increase in CBF in patients who had an impaired haemodynamic state of the brain at presentation, but improvement in CBF was not different between interventional and standard treatment.

Current knowledge of cerebral haemodynamic and metabolic changes over time in medically treated patients with an ICA occlusion is based on small patient series, and both improvement and deterioration over time have been described. In one study, ten medically treated patients with symptomatic ICA occlusion and increased OEF underwent a second PET scan after 12 to 59 months [22]. Over time, patients showed on average an increase in CBF hemispheric (ipsilateral/contralateral) ratio and a decrease in OEF hemispheric ratio, but without changes in absolute values of CBF and OEF [22]. Another study of seven patients with symptomatic ICA occlusion and normal CBF and OEF at baseline found a significant decrease in CBF and increase in OEF after 24 to 64 months, implicating haemodynamic deterioration over time [23]. Other studies reported a bilateral increase in cerebrovascular reactivity measured by Transcranial Doppler (TCD) after CEA of a contralateral carotid stenosis in the presence of a symptomatic or asymptomatic ICA occlusion [9,10,24]. In contrast to these studies, we did not find a significant effect of treatment aimed at improving the flow state of the brain when compared to control patients in a randomised design. A possible explanation could be that the cerebral haemodynamic state also improved without intervention. Patients with haemodynamic compromise at baseline who received standard treatment showed a small non-significant increase in CBF. It has been shown before that patients with ICA occlusion and a compromised flow state of the brain may improve spontaneously over time [25]. In addition, in patients with a normal haemodynamic state of the brain (stage 0) at baseline who underwent an intervention, particularly those treated by tapering of antihypertensive medication, a further increase in CBF may not be expected because of preserved cerebral autoregulation.

A limitation of our randomised pilot study is that we did not select patients on the basis of demonstrated cerebral haemodynamic compromise. The design of the study was aimed at including patients at high risk of recurrent stroke by selection on clinical criteria: ischaemic symptoms of the brain after documented occlusion, excluding patients with eye symptoms alone and those with symptoms only before the ICA occlusion were documented without ongoing symptoms thereafter [26]. We had expected that these clinical criteria would have resulted in a much larger proportion of patients with haemodynamic stages 1 and 2. Because the average time between the last symptoms of patients and the baseline PET scan was 38 days, spontaneous improvement of a compromised flow state of the brain may have occurred before the PET scan took place. This may have contributed to the lack of a difference between the PET scan at baseline and after 3 months.

Another limitation might be heterogeneity in treating patients assigned to intervention. We have chosen for this randomization scheme rather than designing separate studies for each of the treatment options because all treatment options aim to improve cerebral perfusion. However, effects of tapering of antihypertensive medication on the haemodynamic state of the brain may well be different from those of surgery or stenting of stenosed arteries that may be important for collateral blood supply. Subgroup analysis of the present data comparing surgery or stenting of a stenosis in the contralateral ICA, ipsilateral ECA or VA with standard medical treatment alone did not show significant differences in CBF, but the number of patients in each group was small. Likewise, effects of surgery or stenting of the contralateral ICA on cerebral perfusion may be different than those of surgery or stenting of the ipsilateral ECA or VA. Furthermore, it has been described that both endarterectomy and stenting can be complicated by a hyperperfusion syndrome [27], but in none of the patients, this complication occurred.

The third limitation is that a relatively high number of patients had to be excluded because they did not have two successfully performed PET studies. Our failure rate was similar to the rate of 22% for quantitative studies in the St Louis Study [3,6], and lower than in another PET study that reported a failure rate of 41% [28]. Finally, the sample size in our study was probably too small to detect differences between the two treatment strategies except when they were large.

## Conclusions

Although the results of this pilot study are not conclusive, some important lessons can be learned. Patients with TIA or ischaemic stroke in the hemisphere ipsilateral to the ICA occlusion are at risk of subsequent cerebral ischaemia. In these patients with symptomatic ICA occlusion, the clinical benefit of CEA or endovascular treatment of an additional stenosis in the cerebropetal arteries or tapering of antihypertensive medication has not been shown by a randomised trial. The recently published results of the Carotid Occlusion Surgery Study randomised trial [4] that showed no benefit of STA-MCA bypass surgery over medical treatment despite improvement of cerebral haemodynamics in the surgical group reinforce the need for other therapies to improve outcome in patients with ICA occlusion at high risk of recurrent stroke. Oxygen-15 PET is useful to identify a subgroup of patients with haemodynamic compromise, but we could not demonstrate that stenting or surgery of stenosed cerebropetal arteries or tapering of antihypertensive medication improved the haemodynamic state of the brain to a large extent in comparison with standard treatment. Whether revascularization of collaterals or tapering of antihypertensive medication can reduce the risk of recurrent stroke can only be determined in a large randomised trial of patients with a symptomatic ICA occlusion at high risk of stroke.

## Competing interests

The authors declare that they have nothing to declare.

## Authors’ contributions

SP participated in the design of the study, performed the statistical analysis and drafted the manuscript. JPB, BNMB, RB and AAL were involved in the acquisition and interpretation of the PET studies, and critically revised the manuscript. AA performed the statistical analysis and critically revised the manuscript. GJB, LJK and CJMK participated in the design of the study and critically revised the manuscript. All authors read and approved the final manuscript.
